# 17DD Yellow Fever Revaccination and Heightened Long-Term Immunity in Populations of Disease-Endemic Areas, Brazil

**DOI:** 10.3201/eid2508.181432

**Published:** 2019-08

**Authors:** Ana Carolina Campi-Azevedo, Vanessa Peruhype-Magalhāes, Jordana Grazziela Coelho-dos-Reis, Lis Ribeiro Antonelli, Christiane Costa-Pereira, Elaine Speziali, Laise Rodrigues Reis, Jandira Aparecida Lemos, José Geraldo Leite Ribeiro, Luiz Antônio Bastos Camacho, Maria de Lourdes de Sousa Maia, Sheila Maria Barbosa de Lima, Marisol Simões, Reinaldo de Menezes Martins, Akira Homma, Luiz Cosme Cota Malaquias, Pedro Luiz Tauil, Pedro Fernando Costa Vasconcelos, Alessandro Pecego Martins Romano, Carla Magda Domingues, Andréa Teixeira-Carvalho, Olindo Assis Martins-Filho, Collaborative Group for Studies of Yellow Fever Vaccine

**Affiliations:** Instituto René Rachou of Fundação Oswaldo Cruz (FIOCRUZ-Minas),; Belo Horizonte, Brazil (A.C. Campi-Azevedo, V. Peruhype-Magalhāes, J.G. Coelho-dos-Reis, L.R. Antonelli, C. Costa-Pereira, E. Speziali, L.R. Reis, A. Teixeira-Carvalho, O.A. Martins-Filho);; Universidade Federal de Minas Gerais, Belo Horizonte (J.G. Coelho-dos-Reis);; Secretaria Municipal de Saúde, Belo Horizonte (J.A. Lemos);; Secretaria do Estado de Saúde de Minas Gerais, Belo Horizonte (J.G.L. Ribeiro);; Escola Nacional de Saúde Pública (FIOCRUZ-Rio), Rio de Janeiro, Brazil (L.A.B. Camacho);; Instituto de Tecnologia em Imunobiológicos Bio-Manguinhos (FIOCRUZ-Rio),; Rio de Janeiro (M. de Lourdes de Sousa Maia, S.M. Barbosa de Lima, M. Simões, R. de Menezes Martins, A. Homma);; Universidade Federal de Alfenas, Alfenas, Brazil (L.C.C. Malaquias);; Universidade de Brasília, Brasília, Brazil (P.L. Tauil);; Instituto Evandro Chagas, Ananindeua, Brazil (P.F.C. Vasconcelos);; Secretaria de Vigilância em Saúde—Ministério da Saúde, Brasília (A.P.M. Romano, C.M. Domingues)

**Keywords:** yellow fever, 17DD vaccine, neutralizing antibodies, memory CD8^+^ T-cells, vector-borne infections, viruses, Brazil

## Abstract

We evaluated the duration of neutralizing antibodies and the status of 17DD vaccine–specific T- and B-cell memory following primary and revaccination regimens for yellow fever (YF) in Brazil. We observed progressive decline of plaque-reduction neutralization test (PRNT) seropositivity and of the levels of effector memory CD4+ and CD8+ T cells, as well as interferon-γ+CD8+ T cells, 10 years after primary vaccination. Revaccination restored PRNT seropositivity as well as the levels of effector memory CD4+, CD8+, and interferon-γ+CD8+ T cells. Moreover, secondary or multiple vaccinations guarantee long-term persistence of PRNT positivity and cell-mediated memory 10 years after booster vaccination. These findings support the relevance of booster doses to heighten the 17DD-YF–specific immune response to guarantee the long-term persistence of memory components. Secondary or multiple vaccinations improved the correlates of protection triggered by 17DD-YF primary vaccination, indicating that booster regimens are needed to achieve efficient immunity in areas with high risk for virus transmission.

Yellow fever (YF) vaccination is recommended for persons living in YF-endemic areas as the most effective strategy to reduce the risk for infection (*1*). The 17D and 17DD live attenuated vaccines are considered similarly safe and immunogenic, regardless of the minor differences in their nucleotide sequences (*1*). The progressive expansion of areas with YF viral circulation in YF-endemic countries has required extensive vaccination campaigns that reduced the international vaccine stockpile and brought to light the discussion about the need for booster doses to guarantee long-term cell memory in populations living in YF-endemic countries. Outbreaks of YF occur occasionally in areas of Africa and South America (*2*–*7*).

In 2013, the World Health Organization (WHO) stated that a single dose of YF vaccine sufficed to provide lifelong protection and that no booster dose was required to guarantee protection against the disease (*1*,*8*). However, time-dependent loss of protective immunity has been reported (*9*–*11*). The levels of YF-neutralizing antibodies decrease significantly 10 years after vaccination; ≈25%–30% of primary vaccinees lack protective antibodies (*10*,*11*). In addition, the polyfunctional cellular immune responses elicited by YF vaccination that contribute to protection also displayed a time-dependent decline following primary vaccination (*11*,*12*). In light of this information, the single-dose regimen for YF vaccine has been questioned, especially in YF-endemic countries where the proportion of persons exposed to potential risks should be considered against the primary-vaccine failure rate and time-dependent decline of protective immunity.

The goal of this study was to evaluate the proxies of protection elicited by primary, secondary, and multiple vaccinations and verify the duration of neutralizing antibodies and 17DD-specific T- and B-cell memory following these distinct vaccination regimens. We sought to clarify the importance of 17DD-YF booster vaccination to heighten the immune response of those primary vaccinees living in endemic areas whose immunity declines to nonprotective levels.

## Materials and Methods

### Study Population

**Figure 1:**
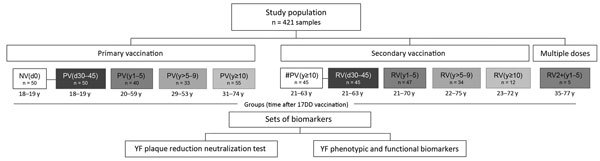
Study population and methods for analyzing 17DD vaccine–specific neutralizing antibodies and phenotypic/functional cell memory in YF. The primary vaccination arm (reference group) includes participants who have never been vaccinated or have had 1 YF vaccination; secondary vaccination arm includes participants who have received 1 or 2 vaccinations; and multiple doses arm includes participants who have received >2 revaccinations. Participant subgroups indicate number of days or years since vaccination (in parentheses; d0 for those never vaccinated). Participant age ranges are given below subgroup boxes. #PV, had primary vaccination >10 years previously; NV, not vaccinated; PV, had primary vaccination only; RV, revaccinated; YF, yellow fever.

We conducted this investigation during May 12, 2014–December 16, 2016, simultaneously sampling from Rio de Janeiro and 2 municipalities of Minas Gerais state (Alfenas and Ribeirão das Neves), Brazil. We assigned participants to groups on the basis of official vaccination records. The study included 421 samples collected from 326 healthy adults 18–77 years of age, initially categorized into 3 arms: primary vaccination, secondary vaccination, and multiple vaccination (Figure 1). We designed the primary vaccination and secondary vaccination arms as 2 complementary independent approaches, each including a longitudinal (95 paired samples) and a cross-sectional investigation (231 unpaired samples). Study groups were coded to indicate participants’ vaccination status (NV for nonvaccinated persons, PV for those who had had primary YF vaccination only, RV for those who had been revaccinated) and time since last vaccination, given in days or years (e.g, d0 for day zero).

### Samples and Tests

We collected whole blood samples from each participant. We used samples of 5 mL without anticoagulant for plaque-reduction neutralization test (PRNT) and samples of 20 mL in heparin for 17DD-YF phenotypic and functional analyses.

#### PRNT

We used serum samples to quantify the PRNT levels to the 17DD-YF virus by the micro-PRNT50 test, as described previously by Simões et al. (*13*). We performed assays at Laboratório de Tecnologia Virológica (LATEV), Bio-Manguinhos, and expressed results from replicates as the reciprocal of sample dilution, considering seropositivity of PRNT titers >1:50 serum dilution.

#### Dengue IgG Indirect ELISA

We performed serologic tests for dengue virus (DENV) IgG using a Panbio dengue IgG indirect ELISA kit (https://www.alere.com). Tests were performed at Laboratório de Flavivírus, Instituto Osvaldo Cruz, as previously reported (*14*).

#### Phenotypic and Functional Memory Biomarkers 

We performed in vitro 17DD-YF–specific peripheral blood lymph proliferative assay as previously reported by Costa-Pereira et al. (*12*). In brief, we incubated replicates of PBMC suspension (1.0 × 10^6^/well) for 144 hours at 37°C in 5% CO_2_. We harvested cells from control (CC) and 17DD-YF antigen–stimulated (17DD-YF Ag) cultures, labeled them with live/dead dye (Life Technologies, https://www.thermofisher.com), and used a cocktail of monoclonal antibodies (mAbs) to quantify the phenotypic memory status of T cells and B cells. For T cells we used anti-CD4/(RPA-T4)/FITC, anti-CD8/(SK1)/PerCP-Cy5.5, anti-CD27/(M-T271)/PE, anti-CD45RO/(UCHL1)/PE-Cy7, and anti-CD3/(SK7)/APC-Cy7; for B cells, we used anti-CD19/(HIB19)/PerCP, anti-CD27/(M-T271)/PE, and anti-IgD/(IA6–2)/FITC. We obtained all mAbs from BD Pharmingen (https://bdbiosciences.com).

In parallel, we stained cultured PBMC aliquots with live/dead dye and a mix of mAbs to quantify the functional memory status of T and B cells: anti-CD3/(UCHT1)/Qdot605 (Invitrogen, https://www.thermofisher.com); anti-CD4/(GK1.5)/APCe-Fluor780 (eBioscience, https://www.thermofisher.com); anti-CD8/(SK1)/PerCP (BD Biosciences, https://bdbiosciences.com); and anti-CD19/(HIB19)/Alexa-Fluor700 (eBioscience). After a fix/permeabilize procedure, we incubated cells with a mAbs cocktail of anti-TNF-α/(clone MAb11)/PE-Cy7, anti-interferon (IFN)-γ/(clone B27)/Alexa-Fluor488), anti-interleukin (IL)-5/(JES1–39D10)/PE, and anti-IL-10/(JES3–19F1)/APC, all from BD Biosciences. We fixed the stained cells and stored them at 4°C for <24 hours before acquisition on a BD LSR Fortessa Flow Cytometer (BD Biosciences).

We acquired a total of 100,000 lymphocytes from each sample. We used FlowJo version 9.3.2 software (Tree Star, https://www.flowjo.com) to quantify the memory T-cell and B-cell subsets, as well as the percentage of cytokine-producing T and B cells. We expressed the results as 17DD-YF Ag/CC Index, calculated as the ratio of cells observed in the 17DD-YF Ag cultures divided by the respective control culture.

### Data Analysis

This study was composed of 2 independent but complementary approaches: a longitudinal investigation and a cross-sectional investigation. We performed statistical analyses for the longitudinal investigation using paired t-test to compare NV(d0) versus PV(d30–45) groups for primary vaccination, as well as #PV(y>10) (primary vaccination >10 years ago) versus RV(d30–45) for secondary vaccination. For the cross-sectional design, we made the transversal comparisons among groups using analysis of variance adjusted to multiple comparisons and set statistical significance at p<0.05. (We did not highlight nonsignificant differences in the figures.) We used the χ^2^ test to compare seropositivity rates between NV and PV groups and also between #PV and RV groups.

We performed biomarker signature analysis as described previously by Luiza-Silva et al. (*15*). In brief, we calculated the global median value of 17DD-YF Ag/CC Index for each phenotypic and functional biomarker and used that value as the cutoff to identify each biomarker as low index (below global median) or high index (above global median). We considered only biomarkers observed in >50% of study participants for comparative analysis among groups.

We conducted Venn diagram analysis (http://bioinformatics.psb.ugent.be/webtools/Venn) to select common biomarkers among subgroups. We overlaid biomarker signatures for comparative analysis of time-dependent changes of biomarker sets observed after vaccination.

## Results

### Booster Vaccination and PRNT 

**Figure 2:**
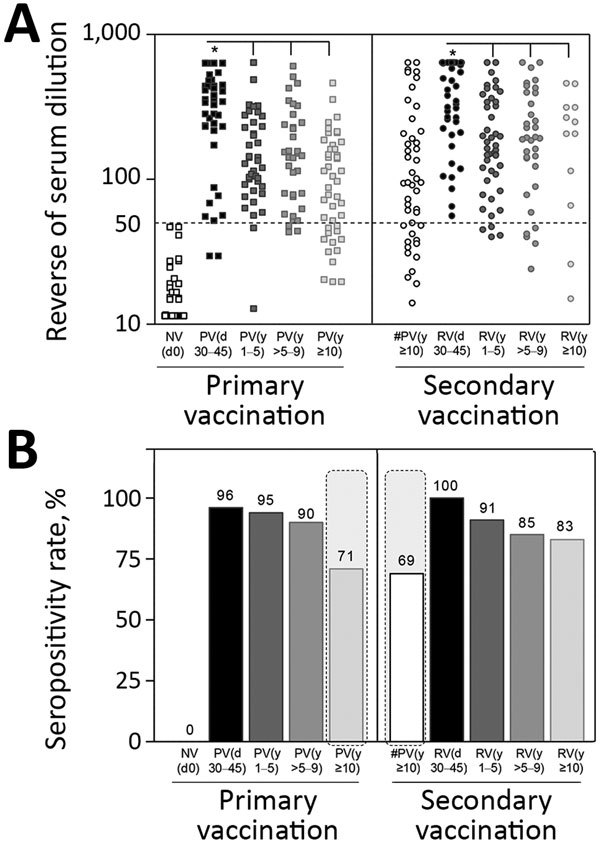
Neutralizing antibody levels and seropositivity rates before and after primary and secondary 17DD vaccination for YF. We detected 17DD-specific neutralizing antibodies by micro plaque-reducing neutralization test (micro-PRNT50) and determined seropositivity rates by considering serum dilution >1:50 as the cutoff criterion for PRNT positivity. A) Scatter graph of PRNT titers, expressed as reverse of serum dilution. B) Percentage of PRNT seropositivity (serum dilution >1:50). Gray dashed lines indicate critical seropositivity rates <80%. Participant subgroups indicate number of days or years since vaccination (in parentheses; d0 for those never vaccinated). #PV, had primary vaccination >10 years previously; NV, not vaccinated; PV, had primary vaccination only; RV, revaccinated; YF, yellow fever..

Data analysis demonstrated that primary vaccination triggered significant levels of 17DD-YF–specific neutralizing antibodies (p<0.0001), reaching a seropositivity rate of 96% (Figure 2). Of note, we observed progressive decrease in PRNT levels (p<0.0001) and in the PRNT seropositivity rates along the time compared with PV(d30–45). The seropositivity rate declined to ≈71% by 10 years after primary vaccination (Figure 2, panel B). Booster vaccination significantly increased the PRNT levels (p<0.0001) and raised the seropositivity rate from 69% to 100% (Figure 2, panels A, B). The secondary vaccination was accompanied by higher seropositivity rate after 10 years upon booster dose, regardless of the decrease in PRNT levels (p<0.0001) observed over time compared with #PV(d30–45) (Figure 2, panel B).

### Secondary Booster Vaccination and YF-specific Cell-Mediated Memory 

**Figure 3:**
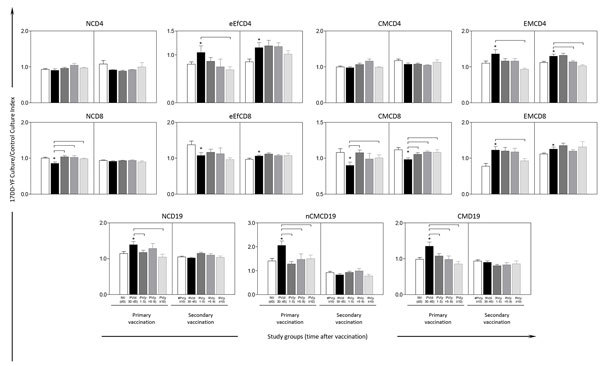
Phenotypic memory biomarkers before and after primary and secondary 17DD vaccination for YF. Distinct T-cell memory subsets included naive T cells/(NCD4;NCD8)/CD27+CD45RO–; early effector memory T cells/(eEfCD4;eEfCD8)/CD27–CD45RO–; central memory T cells/(CMCD4;CMCD8)/CD27+CD45RO+ and effector memory T cells/(EMCD4;EMCD8)/CD27–CD45RO+. B-cell memory subsets included naive B cells/(NCD19)/CD27–IgD+; nonclassical memory B cells/(nCMCD19)/CD27+IgD+ and classical memory B cells/(CMCD19)/CD27+IgD–.We performed intra-arm analyses by paired t-test to compare memory-related phenotypic features observed in participants with previous vaccination with paired samples collected early after vaccination: NV(d0) versus PV(d30–45) and #PV(y≥10) versus RV(d30–45). Asterisks (*) indicate significant differences (p<0.05); vertical error bars indicate SEM. For intergroup analysis, we used adjusted analysis of variance to compare the memory-related phenotypic features observed among distinct time points; bars connecting subgroups indicate significant differences (p<0.05) between postvaccination subgroups. Participant subgroups indicate number of days or years since vaccination (in parentheses; d0 for those never vaccinated).

Comparative analysis of NV(d0) versus PV(d30–45) after 1 or 2 doses of 17DD-YF vaccine demonstrated that primary vaccination is followed by an increase of memory T cells, including eEfCD4 (p<0.05), EMCD4 (p<0.05), and EMCD8 (p = 0.0006), and all B-cell subsets evaluated, NCD19 (p = 0.01), nCMCD19 (p = 0.001), and CMCD19 (p = 0.001). We also observed a decrease of NCD8 (p = 0.02), eEfCD8 (p = 0.02), and CMCD8 (p = 0.001). The results showed that cellular immunity, eEfCD4 (p = 0.01), EMCD4 (p = 0.01), and EMCD8 (p = 0.009); and all B-cell subsets, NCD19 (p = 0.003), nCMCD19 (p = 0.02), and CMCD19 (p = 0.0002), clearly wane over 10 years, compared with 30–45 days after primary vaccination (Figure 3).

Secondary vaccination with 17DD-YF was able not only to increase the level of memory T-cell subsets, eEfCD4 (p<0.05), EMCD4 (p<0.05), and EMCD8 (p = 0.04) at 30–45 days after the second dose but also to sustain the maintenance of eEfCD4 and EMCD8 levels even after >10 years upon booster vaccination compared with 30–45 days after secondary vaccination. We observed no substantial changes in memory B-cell subsets upon secondary vaccination (Figure 3).

### Revaccination and IFN-γ–Mediated T-Cell Memory

**Figure 4:**
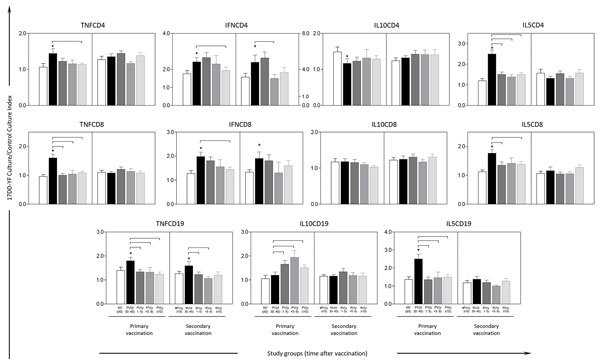
Functional biomarkers specific to 17DD vaccine for YF before and after primary and secondary vaccination. We performed analysis upon in vitro 17DD-YF antigen recall of peripheral blood mononuclear cells. Flow cytometric immunophenotypic staining were performed to quantity functional T-cell subsets producing TNF-α, IFN-γ, IL-10, and IL-5 and B-cell subsets producing TNF-α, IL-10, and IL-5. We performed intra-arm analyses by paired t-test to compare memory-related functional features observed in previously vaccinated participants with paired samples collected early after vaccination. Asterisks (*) indicate significant differences (p<0.05); vertical error bars indicate SEM. For intergroup analysis, we used adjusted analysis of variance to compare the memory-related phenotypic features observed among distinct time points; connecting lines indicate significant differences. Participant subgroups indicate number of days or years since vaccination (in parentheses; d0 for those never vaccinated). IFN, interferon; IL, interleukin; TNF, tumor necrosis factor; YF, yellow fever.

Data analysis for in vitro 17DD-YF antigen recall revealed that primary vaccination induced significant increases of functional memory biomarkers in CD4+ (tumor necrosis factor [TNF]–α/p = 0.04, IFN-γ/p = 0.04 and IL-5/p = 0.0001) and CD8+ T cells (TNF-α/p = 0.0003, IFN-γ/p = 0.008 and IL-5/p = 0.0002) as well as in B cells (TNF-α/p<0.05 and IL-5/p = 0.016) (Figure 4). We observed a decrease of IL-10+CD4+ T cells (p = 0.04) at 30–45 days after primary vaccination and a clear decrease of TNF-α, IFN-γ, and IL-5 produced by CD4+ and CD8+ T cells. We also saw a decrease in TNF-α and IL-5 from B cells over time, particularly at >10 years, compared with 30–45 days after primary vaccination (p<0.05 in all cases). Conversely, we detected an increase of IL-10+ B cells over time after primary vaccination (Figure 4).

Secondary vaccination was able to restore T cell functional memory mediated by IFN-γ and B-cell functional memory by TNF-α. We observed sustained production of IFN-γ by CD8+ T cells even >10 years after booster vaccination (Figure 4).

### Booster Vaccination and Long-lasting Persistence of Effector Memory 

We constructed overlays of biomarker signatures of NV(d0) versus PV(d30–45) in the primary-vaccination arm of the study as well as #PV(y>10) versus RV(d30–45) in the secondary-vaccination arm to select those attributes eligible as universal memory-related biomarkers (Appendix Figure 1). Venn diagram analysis revealed 3 common attributes (EMCD4, EMCD8, and IFNCD8) that we tagged for follow-up analysis after primary, secondary, or multiple 17DD-YF vaccination (Appendix Figure 1).

**Figure 5:**
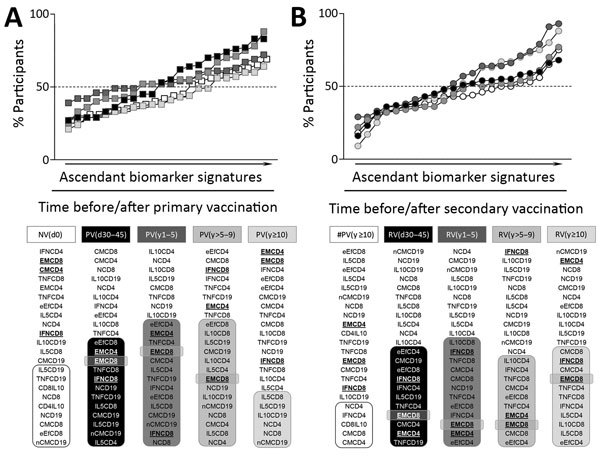
Ascendant biomarker signatures before and after primary and secondary 17DD vaccination for YF. Overlaid biomarker signatures were assembled to identify changes in the 17DD-specific phenotypic and functional features observed over time in primary vaccination arm (A) and secondary vaccination arm (B). Shading indicates time point before and after primary or secondary vaccination for each biomarker; dashed line indicates the global median >50th percentile. Boldface text indicates the 3 biomarkers considered relevant universal attributes to monitor 17DD-YF–specific memory (EMCD4, EMCD8, IFNCD8). Participant subgroups indicate number of days or years since vaccination (in parentheses; d0 for those never vaccinated). IFN, interferon; IL, interleukin; TNF, tumor necrosis factor; YF, yellow fever.

After we selected the universal set of follow-up attributes, we compared biomarker signatures over time after 17DD-YF primary or secondary vaccination (Figure 5). Data analysis demonstrated that all 3 biomarkers were observed in PV(d30–45) and PV(y1–5). EMCD8 was maintained in PV(y>5–9) but not in PV(y>10). Comparative analysis between #PV(y>10) and RV(d30–45) demonstrated restoration of universal memory-related biomarkers (EMCD4, EMCD8, and IFNCD8) upon revaccination. Moreover, we identified EMCD8 in a high proportion of secondary vaccinees across the time periods after booster dose (Figure 5).

### Comparison of 17DD-YF Primary and Booster Vaccination Effects on Cell-Mediated Memory 

**Figure 6:**
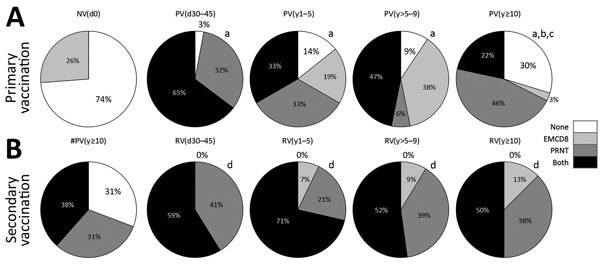
Overall proportion of participants with resultant memory before and after primary or secondary 17DD vaccination for YF as assessed by PRNT and EMCD8 measurement at participant level. Graphs show the proportion of participants above the cutoff threshold; that is, PRNT positivity at serum dilution >1:50 and EMCD8 index above the global median value. We then determined the resultant memory for each subgroup in the primary vaccination arm (A) and secondary vaccination (B) of the study. Participant subgroups indicate number of days or years since vaccination (in parentheses; d0 for those never vaccinated). Lowercase letters indicate significant differences (p<0.05 by χ^2^ test) of resultant memory status among study groups: a for comparisons with NV(d0) participants; b for comparisons with PV(d30–45) participants; c for comparison with PV(y1–5) participants; and d for comparison with #PV(y>10) participants. #PV, had primary vaccination >10 years previously; NV, not vaccinated; PRNT, plaque-reducing neutralization test; PV, had primary vaccination only; RV, revaccinated; YF, yellow fever.

We used individual PRNT and EMCD8 profiles to assemble a memory matrix and calculated the resultant YF-specific memory. We classified each volunteer by positive results above the cutoff threshold as EMCD8, PRNT, none, or both (Figure 6). Data analysis demonstrated that primary vaccination leads to a resultant memory in 97% of volunteers, with 3% of failure in the PV(d30–45) group. However, an increase in the proportion of primary vaccinees with “none” positive attributes, neither PRNT nor EMCD8 biomarkers, was observed, reaching a critical value of 30% at ≥10 years after primary vaccination (Figure 6, panel A).

The comparison between #PV(y>10) and RV(d30–45) demonstrated that secondary vaccination restored the resultant memory in 100% of the volunteers, which was different from primary vaccination results. All secondary vaccines evaluated simultaneously for PRNT and EMCD8 profile presented a preserved resultant memory. In particular, at >10 years after secondary vaccination, 100% of vaccinees had 1 or both biomarkers detectable (Figure 6, panel B).

### Multiple Vaccination and the Overall Profile of Protection 

**Figure 7:**
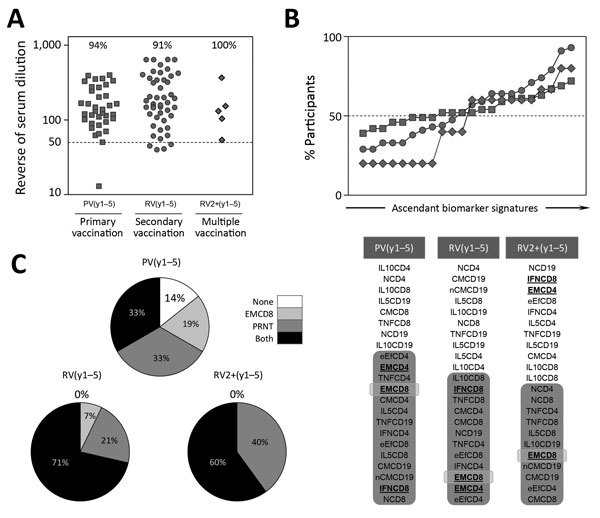
Overall profile of immune response after primary, secondary, or multiple 17DD vaccination for YF. A) Levels of 17DD-YF–specific neutralizing antibodies; B) 17DD-YF–specific phenotypic and functional biomarkers; and C) resultant memory status PRNT and EMCD8 measurement (PRNT and EMCD8) for individual participants. Results are expressed in reverse of serum dilution, percentage of participants with overlaid biomarker signatures, and resultant memory status at 1–5 years after primary (gray circle), secondary (gray square), or multiple (gray diamond) vaccination. Participant subgroups indicate number of days or years since vaccination (in parentheses; d0 for those never vaccinated). #PV, had primary vaccination >10 years previously; IFN, interferon; IL, interleukin; NV, not vaccinated; PRNT, plaque-reducing neutralization test; PV, had primary vaccination only; RV, revaccinated; TNF, tumor necrosis factor; YF, yellow fever.

We analyzed 17DD-YF memory status triggered by multiple vaccinations (Figure 7). The results demonstrated that all vaccinees who received multiple shots had PRNT levels above the cutoff limit (Figure 7, panel A). The proportion of vaccinees with EMCD8 above the cutoff limit was higher for RV(y1–5) and RV2+(y1–5) than for PV(y1–5) (Figure 7, panel B). Likewise, the overall resultant memory for RV(y1–5) and RV2+(y1–5) was higher than that for PV(y1–5) – (Figure 7, panel C).

### PRNT Seronegativity before Revaccination and Cell-Mediated Memory Response 

**Figure 8:**
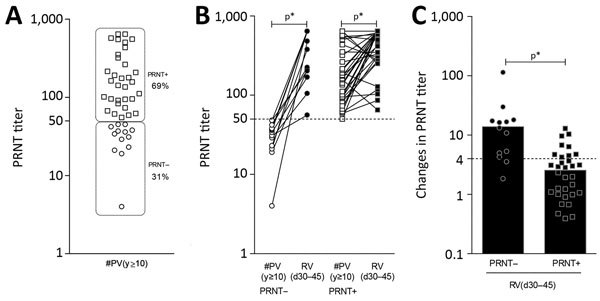
Baseline PRNT reactivity before revaccination (A) and impact on PRNT levels triggered by secondary 17DD vaccination for YF (B, C). Subgroups PRNT– (circles) and PRNT+ (squares) were defined considering the cutoff criterion for PRNT positivity at serum dilution >1:50. The ability of secondary vaccination to increase the levels of neutralizing antibodies as well as the magnitude of changes in PRNT titers (baseline fold changes) are indicated for PRNT– (filled circles) and PRNT+ (filled squares) vaccinees. Increases in PRNT titer by a factor of >4 at follow-up were considered as classical criteria to evaluate booster response. Bars indicate significant differences (p<0.05) between subgroups. Participant subgroups indicate number of days or years since vaccination (in parentheses). #PV, had primary vaccination >10 years previously; PRNT, plaque-reducing neutralization test; RV, revaccinated; YF, yellow fever.

**Figure 9:**
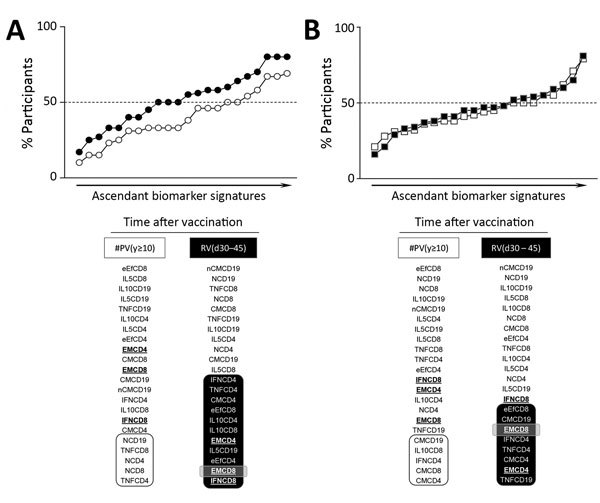
Impact of baseline plaque-reducing neutralization test (PRNT) reactivity on memory-related biomarkers triggered by secondary 17DD vaccination for YF. We assessed the impact of secondary vaccination on phenotypic and functional biomarkers in participants with negative (A) and positive (B) PRNT results. Shading indicates time point after primary vaccination for each biomarker: white for participants who were vaccinated >10 years previously, and black for those who were revaccinated in the previous 30–45 days. The dashed line shows the global median >50th percentile. Boldface text indicates the 3 biomarkers considered relevant universal attributes to monitor 17DD-YF–specific memory (EMCD4, EMCD8, IFNCD8). Participant subgroups indicate number of days or years since vaccination (in parentheses). #PV, had primary vaccination >10 years previously; IFN, interferon; IL, interleukin; RV, revaccinated; TNF, tumor necrosis factor; YF, yellow fever.

The volunteers from the #PV(y≥10) group were further categorized into subgroups, PRNT– and PRNT+, according to their PRNT results before revaccination. The levels of humoral and cellular biomarkers, as well as the magnitude of baseline fold changes in neutralizing antibodies, were higher in PRNT– than in PRNT+ vaccines (Figures 8, 9). An increase in PRNT titer by a factor >4 at follow-up further demonstrated the relevance of booster doses to restore the immunological memory of these subjects (Figure 8). Furthermore, the booster dose had higher impact on cellular immunity and biomarker signature of PRNT– than PRNT+ primary vaccinees, and both groups restored the EMCD8 biomarker compared with the #PV(y>10) group (Figure 9).

### Dengue Virus Seropositivity

We analyzed the results to determine whether DENV seropositivity influences the humoral and cellular memory after secondary vaccination (Appendix Figures 2, 3). We found DENV seropositivity in 28% of volunteers in the secondary vaccination arm. The results demonstrated that DENV seropositivity did not influence PRNT seropositivity over time after secondary vaccination (Appendix Figure 2). Moreover, DENV seropositivity did not affect cellular immunity (EMCD8), which remained above the cutoff limit in all subgroups of secondary vaccinees (Appendix Figure 3).

## Discussion

YF vaccination induces an efficient immunity and represents one of the most effective strategies to reduce the risk for infection in YF-endemic countries. The vaccine is highly immunogenic, eliciting a strong antibody response together with a broad and complex innate (*16*–*22*) and adaptive immunity (*23*–*28*). Although the YF vaccine has been considered a benchmark among vaccines because of its ability to induce long-lasting immune response, the duration of humoral and cellular immunity following YF vaccination is still controversial. Some studies demonstrate that neutralizing antibodies and YF-specific CD8+ T cells after primary vaccination are suggestive imprints compatible with long-lived memory (*8*,*26*,*29*–*31*); other studies emphasize that the immunity to YF vaccine wanes over time, suggesting the need for booster doses to guarantee long-lasting, efficient immunity memory (*9*–*12*,*32*–*36*).

We could not assess protective immunity to YF in humans by challenge with live wild-type YF virus. Therefore, the protective or nonprotective immunity status to YF virus in humans is based on laboratory methods, known as correlates of protection; PRNT level has been considered the standard for measuring postvaccination immunity to YF. Pinpointing cellular immunity biomarkers is relevant in studies that pose the question of whether PRNT seronegativity necessarily means absence of protective immunity (*34*). Our investigation proposes that, in addition to PRNT seropositivity, YF-specific cellular immunity may be a useful tool for monitoring the duration of YF vaccine-induced memory over time. However, upon closer examination, our findings indicate the decline of YF-specific immune response over time, emphasizing that the YF-specific immunity wanes shortly after primary vaccination and that a substantial proportion of primary vaccines (14%–30%) do not present sufficient levels of neutralizing antibodies or CD8+ T-cell memory within 5–10 years after primary vaccination.

Some studies have investigated the relevance of booster doses to heighten the YF-specific immune response in primary vaccinees whose immunity has declined to nonprotective levels. Weiten et al. (*31*) postulated that booster vaccination did not increase the titers of YF-specific antibodies nor induce or alter the phenotypes of CD8+ T cells. Conversely, Kongsgaard et al. (*37*) have demonstrated that, although most vaccinees responded to a booster vaccination, the antibody titers and the cellular immune responses observed following revaccination were lower than for primary vaccination responses. In this study, we have demonstrated that the booster dose was able to upregulate the levels of neutralizing antibodies and heighten the cellular immunity signature and restore the proportion of vaccinated participants with high levels of effector memory CD8+ T cells. We strongly believe that the differences we observed among the published studies could be attributed to differences in the populations under scrutiny. 

The magnitude of increase in the neutralizing antibody titers and YF-specific CD8+ T-cell response achieved with a booster dose may be closely related to the baseline immune activation, suggesting that an activated immune microenvironment before revaccination impairs the response to the YF vaccine. Muyanja et al. (*38*) showed that 17D-204 vaccinees in Africa displayed decreased levels of YF-specific immunity compared with vaccinees in Europe. It is possible that the immune microenvironment affects the quantitative and qualitative response, as well as the vaccine efficacy. Whereas vaccinees in Europe displayed persistent PRNT levels even at 10 years after vaccination, those in Africa exhibited reduced persistence of YF immunological memory. Constant exposure to infectious diseases, as well as diet and gut microbiota, may lead to a state of immune hyperactivation and exhaustion that can contribute to reduced magnitude of cellular and humoral responses and impair the YF vaccine memory. Of note, the impaired YF vaccine-induced memory was boosted by a second vaccination (*38*). Our results corroborate this hypothesis, showing the relevance of a booster dose to improve the immune response among primary vaccinees. This recommendation should be considered to restore the YF protective immunity in those primary vaccinees whose correlates of protection fade over time, reaching nonprotective levels. 

The loss of immunity in a subpopulation of vaccine recipients should be taken into consideration, and a booster dose should be administered. Previous studies have demonstrated that, on average, approximately 1 in 5 persons from non–YF-endemic areas and 3 in 10 persons living in YF-endemic areas may lose measurable antibody responses within 10 years after primary vaccination. In this sense, it is true that the 17DD-YF vaccination elicits long-lasting immunity; however, lifelong immunity is not observed in every vaccinated individual. Although PRNT is the standard assay to monitor YF protective immunity, it is not a feasible method available in local laboratories to identify participants who should receive a booster vaccination. 

The expansion of risk areas for YF worldwide has contributed to the depletion of YF vaccine stockpile and the need for new strategies to reverse the imminent shortage of vaccine. WHO has considered measures to improve YF vaccine supply, including a dose-sparing strategy as a short-term measure. However, the dose sparing is not proposed for routine immunization. WHO also considered advising a single lifetime dose of YF vaccine. In YF outbreaks or periods of limited vaccine production and reduced stockpiles, the YF primary vaccination should be the priority, as recommended by the Strategic Advisory Group of Experts on Immunization. However, once the YF vaccine supply has normalized and no outbreaks are reported, revaccination should be suggested. On the basis of our findings, >1 booster dose at 10 years after primary vaccination is suggested for travelers entering higher-risk areas and required for residents of YF-endemic countries who are at risk for infection (*34*,*39*).

Altogether, our findings emphasize the relevance of booster doses to heighten the 17DD-YF specific immune response to achieve efficient immunity. Secondary vaccination improved the correlates of protection that had waned over time after 17DD-YF primary vaccination and lowered the loss of protection over time. However, multiple vaccination seems to better support long-lasting protection in all vaccinees, which suggests that >3 booster doses at 10-year intervals should be recommended, especially in areas with high risk for YF transmission. Whether the waning of immune markers observed over time is associated with loss of YF vaccine effectiveness and increased risk for breakthrough YF infection remains to be seen.

In summary, we evaluated the evidence for benefits and risks associated with YF vaccine booster doses using the Grading of Recommendations, Assessment, Development, and Evaluation framework (*40*). A primary dose of YF vaccine is well known to be highly safe and effective, with few vaccine failures. However, a critical wane of correlates of protection has been documented by our group and others, particularly at ≥10 years postvaccination. Few reports of serious adverse events have been observed following booster doses of the vaccine. We recommend the booster dose to prevent this serious disease that has no treatment and substantial mortality rates.

Appendix Additional information about yellow fever revaccination and long-term cell memory, Brazil.
