# Supplementary material for: 17DD Yellow Fever Revaccination and Heightened Long-Term Immunity in Populations of Disease-Endemic Areas, Brazil
Source: Emerg Infect Dis. 2019 Aug;25(8):1511–21. doi: 10.3201/eid2508.181432 (PMC6649311; doi:10.3201/eid2508.181432)
Supplement: Appendix — Additional information about yellow fever revaccination and long-term cell memory, Brazil. [file 18-1432-Techapp-s1.pdf]

Article DOI: https://doi.org/10.3201/eid2508.181432

17DD Yellow Fever Revaccination and Heightened Long-Term Immunity in Populations of Disease-Endemic Areas, Brazil

Appendix

Additional Methods

We collected a total of 421 blood samples from 326 healthy men and women, 18–77 years of age. We categorized participants into 3 study arms: primary vaccination (reference groups), secondary vaccination, and multiple vaccination, on the basis of their status of having had a single dose of 17DD-YF vaccine, secondary (booster) dose, or multiple (>2) vaccination. We sub-grouped participants according to distinct time-point before or after vaccination. The primary vaccination arm comprises 233 samples collected from 183 healthy adults of both sexes. The secondary vaccination arm comprises 183 samples collected from 138 healthy adults of both sexes. The multiple vaccination arm comprises 5 samples collected from 5 healthy adults of both sexes.

We analyzed 17DD-YF–specific memory by establishing the phenotypic features of peripheral blood mononuclear cells upon 17DD-YF antigen recall in vitro. We performed flow cytometric immunophenotypic staining to identify distinct T-cell memory subsets, including naive T cells/(NCD4;NCD8)/$CD27^+CD45RO^-$; early effector memory T cells/(eEfCD4;eEfCD8)/$CD27^-CD45RO^-$; central memory T cells/(CMCD4;CMCD8)/$CD27^+CD45RO^+$ and effector memory T cells/(EMCD4;EMCD8)/$CD27^-CD45RO^+$. B-cell memory subsets were also evaluated and included: naive B cells/(NCD19)/$CD27^-IgD^+$; non-classical memory B cells/(nCMCD19)/$CD27^+IgD^+$ and classical memory B cells/(CMCD19)/$CD27^+$IgD.

We conducted analysis of 17DD-YF–specific functional biomarkers upon in vitro 17DD-YF antigen recall of peripheral blood mononuclear cells. Flow cytometric immunophenotypic staining were performed to quantify functional T-cell subsets producing TNF-α, IFN-γ, IL-10, and IL-5, and B-cell subsets producing TNF-α, IL-10, and IL-5.

For both the memory and biomarker analyses, data were reported as 17DD-YF Ag/CC Index. We performed first intra-arms analyses by paired T-test to compare the memory-related phenotypic features observed before vaccination with paired samples collected early after vaccination NV(d0) vs. PV(d30–45) and #PV(y≥10) vs. RV(d30–45). Additionally, we conducted intergroup analysis by ANOVA adjusted to multiple comparisons to compare the memory-related phenotypic features observed among distinct time-points: PV(d30–45) vs. PV(y1–5) vs. PV(y>5–9) vs. PV(y≥10), as well as RV(d30–45) vs. RV(y1–5) vs. RV(y>5–9) vs. RV(y≥10).

In the comparative analyses between distinct time-points we paid special attention to the set of 3 phenotypic and functional biomarkers considered relevant universal attributes to monitor 17DD-YF specific memory: EMCD4, EMCD8, and IFNCD8. Particular attention was given to EMCD8, previously reported by Costa-Pereira et al. (2018) as one of the top biomarkers to monitor the immunological memory to 17DD-YF vaccine.

We assessed resultant memory by plaque-reducing neutralization test (PRNT) and EMCD8 measurement before and after primary and secondary 17DD-YF vaccination at participant level to calculate the overall proportion of participants for whom test results showed EMCD8 or PRNT levels above the cutoff threshold, i.e., PRNT positivity at serum dilution >1:50 and EMCD8 index above the global median value (17DD-YF Ag/CC Index >1.13). Then, we performed χ^2 tests to determine the resultant memory for each subgroup in the primary vaccination and secondary vaccination study arms. We noted significant differences at $p<0.05$.

We assessed immune response before and after primary, secondary, or multiple 17DD-YF vaccination, including the levels of 17DD-YF–specific neutralizing antibodies, 17DD-YF–specific phenotypic and functional biomarkers, and resultant memory status.

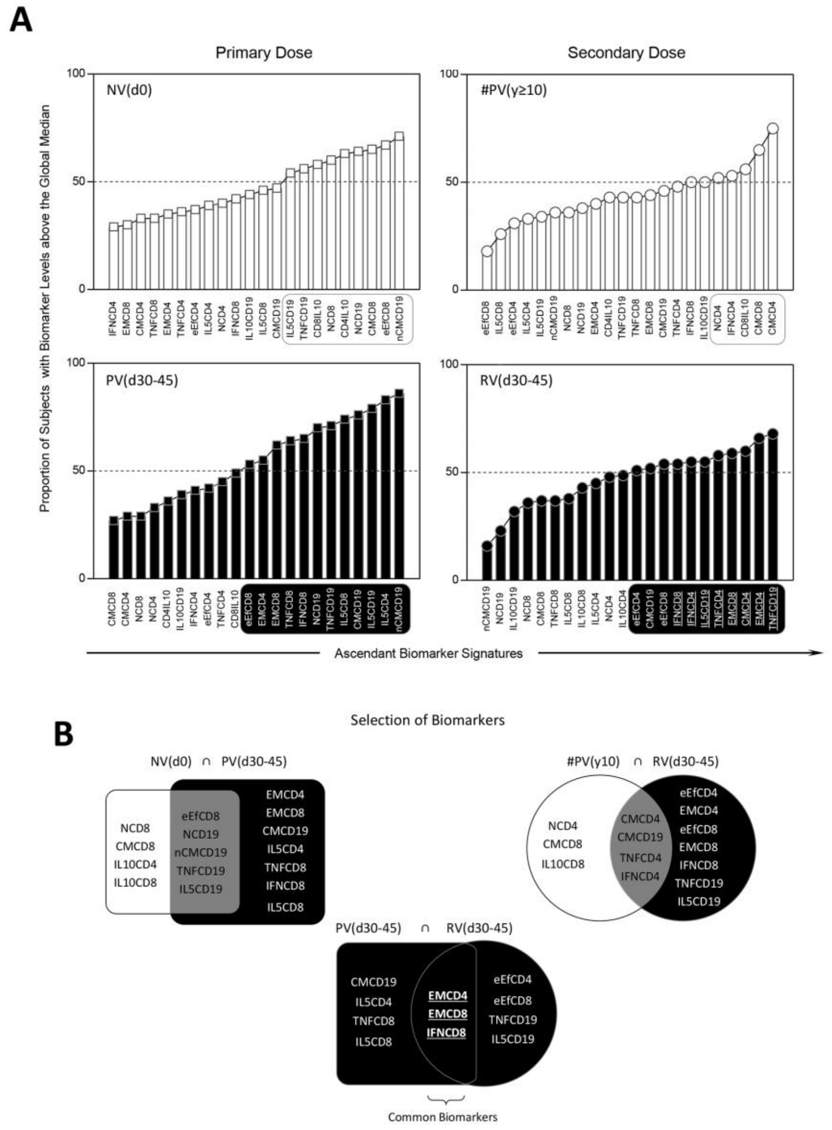

Appendix Figure 1. Selection of memory-related biomarkers up-regulated before and after primary or secondary 17DD-YF vaccination. Participant subgroups indicate number of days or years since vaccination (in parentheses; d0 for those never vaccinated). (A) Ascendant biomarker signatures of NV(d0) (white square) and PV(d30–45) (black square) in the Primary Vaccination arm as well as #PV(y≥10) (white circle) and RV(d30–45) (black circle) in the Secondary Vaccination arm were assembled to select those biomarkers with proportion of subjects above the global median higher than the 50th percentile in each group. (B) Venn diagram analysis were employed to select the set of biomarkers selectively increased in [PV(d30–45) vs. NV(d0)] and in [RV(d30–45) vs. #PV(y≥10)]. Thereafter, additional intersection diagram was constructed to select the common biomarkers observed in PV(d30–45)∩RV(d30–45). The attributes EMCD4, EMCD8 and IFNCD8 were then underscored as universal memory-related biomarkers. These attributes were tagged in bold underline format and employed for follow-up analysis overtime after primary, secondary, or multiple 17DD-YF vaccination.

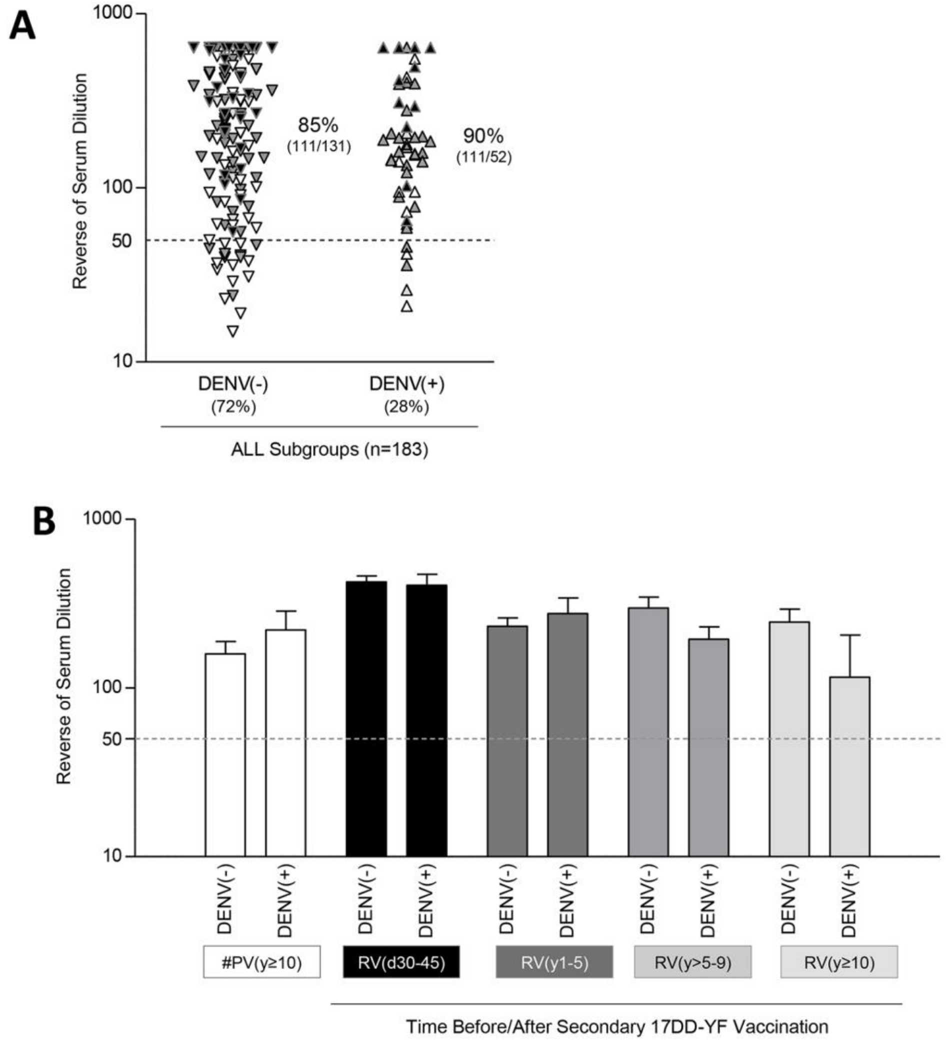

Appendix Figure 2. Neutralizing antibody levels before and after secondary17DD-YF vaccination according to a-DENV IgG reactivity. A) Global ranges and seropositivity rates of neutralizing antibodies in subgroups referred as DENV(–) (inverted triangle) and DENV(+) (triangle) were defined considering the seropositivity for anti-DENV IgG, corresponding to 72% negative and 28% positive in the study population. B) Average of PRNT levels according to the time before or after secondary vaccination: white, #PV(y≥10); black, RV(d30–45); dark gray, RV(y1–5); medium gray, RV(y>5–9); and light gray, RV(y≥10). The results are expressed as reverse of serum dilution and seropositivity rates determined considering the serum dilution higher 1:50 as the cutoff criterion for PRNT positivity. Participant subgroups indicate number of days or years since vaccination (in parentheses; d0 for those never vaccinated).

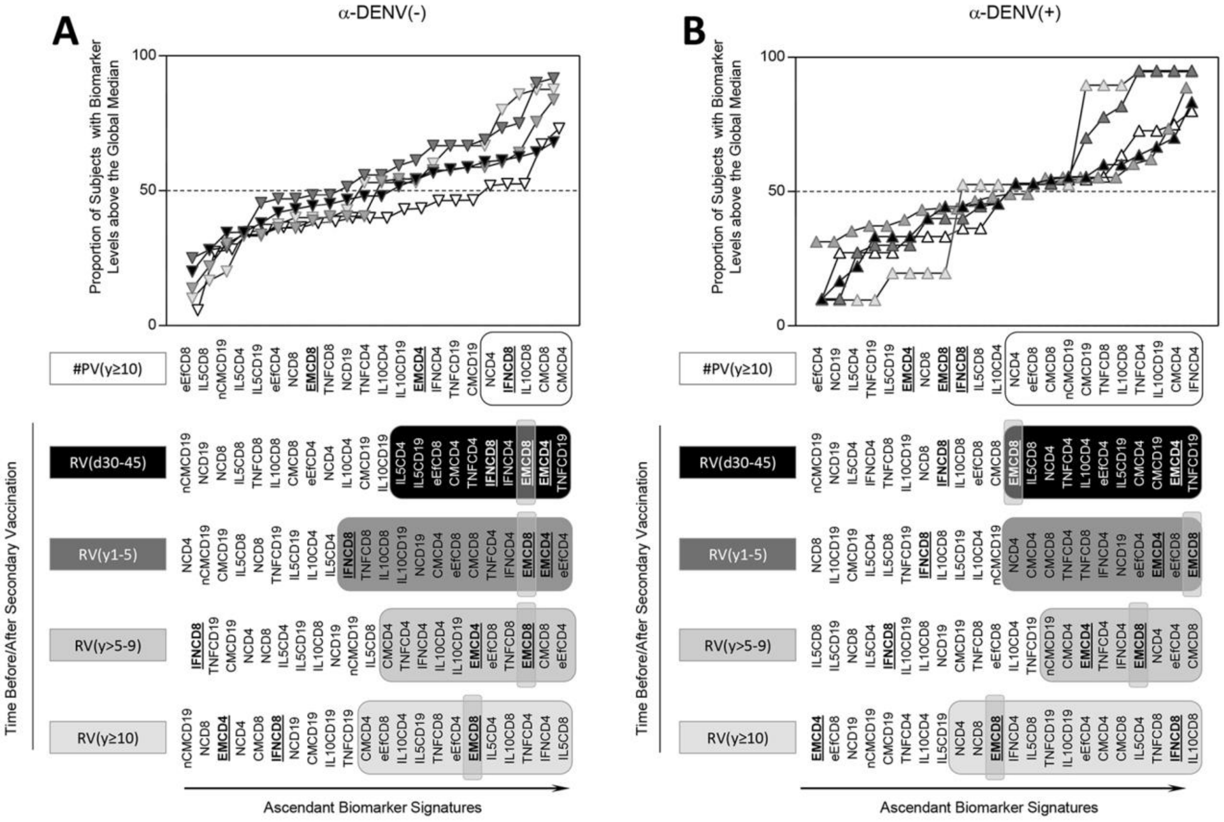

Appendix Figure 3. Ascendant biomarker signatures before and after secondary 17DD-YF vaccination according to a-DENV IgG reactivity. Overlaid biomarker signatures were assembled to identify changes in the 17DD-YF–specific phenotypic and functional features observed over time upon secondary vaccination in subgroups referred as DENV(–) (inverted triangle) and DENV(+) (triangle) were defined considering the seropositivity for anti-DENV IgG. The biomarkers with proportion of subjects above the global median higher than the 50th percentile were underscored by black/grayscale background for DENV(–) and DENV(+) subgroups, at each time point before and after secondary vaccination: #PV(y≥10) = ▽,△; RV(d30–45) = ▼,▲; RV(y1–5) = ▼,▲; RV(y>5–9) = ▽,△ and RV(y≥10) = ▽,△. Participant subgroups indicate number of days or years since vaccination (in parentheses; d0 for those never vaccinated). Comparative analyses between distinct time-points were carried out especially considering the set of 3 phenotypic and functional biomarkers considered relevant universal attributes to monitor 17DD-YF specific memory (EMCD4, EMCD8, IFNCD8, shown in boldface text.
